# The digitized chronic disease management model: scalable strategies for implementing standardized healthcare and big data analytics in Shanghai

**DOI:** 10.3389/fdata.2023.1241296

**Published:** 2023-08-24

**Authors:** Mengyun Sui, Minna Cheng, Sheng Zhang, Yuheng Wang, Qinghua Yan, Qinping Yang, Fei Wu, Long Xue, Yan Shi, Chen Fu

**Affiliations:** ^1^Division of Chronic Non-communicable Diseases and Injury, Shanghai Municipal Center for Disease Control and Prevention, Shanghai, China; ^2^School of Public Health, Fudan University, Shanghai, China; ^3^Medical Department, Huashan Hospital, Fudan University, Shanghai, China

**Keywords:** chronic disease management, Integrated Community Multimorbidity Care Model, digital technology, big data, Shanghai

## Abstract

**Background:**

Chronic disease management (CDM) falls under production relations, and digital technology belongs to the realm of productivity. Production relations must adapt to the development of productivity. Simultaneously, the prevalence and burden of chronic diseases are becoming increasingly severe, leveraging digital technology to innovate chronic disease management model is essential.

**Methods:**

The model was built to cover experts in a number of fields, including administrative officials, public health experts, information technology staff, clinical experts, general practitioners, nurses, metrologists. Integration of multiple big data platforms such as General Practitioner Contract Platform, Integrated Community Multimorbidity Management System and Municipal and District-Level Health Information Comprehensive Platform. This study fully analyzes the organizational structure, participants, service objects, facilities and equipment, digital technology, operation process, etc., required for new model in the era of big data.

**Results:**

Based on information technology, we build Integrated Community Multimorbidity Care Model (ICMCM). This model is based on big data, is driven by “technology + mechanism,” and uses digital technology as a tool to achieve the integration of services, technology integration, and data integration, thereby providing patients with comprehensive people-centered services. In order to promote the implementation of the ICMCM, Shanghai has established an integrated chronic disease management information system, clarified the role of each module and institution, and achieved horizontal and vertical integration of data and services. Moreover, we adopt standardized service processes and accurate blood pressure and blood glucose measurement equipment to provide services for patients and upload data in real time. On the basis of Integrated Community Multimorbidity Care Model, a platform and index system have been established, and the platform's multidimensional cross-evaluation and indicators are used for management and visual display.

**Conclusions:**

The Integrated Community Multimorbidity Care Model guides chronic disease management in other countries and regions. We have utilized models to achieve a combination of services and management that provide a grip on chronic disease management.

## Introduction

### Rationale for strengthening digital technology

Internet of things, health-related internet, digital device, apps, and other digital technology fall under the productivity category (Gunasekeran et al., 2021; Ng et al., 2021). Chronic disease management (CDM) and CDM models are the domain of productive relationships. The productive relationships must be adapted to the growth of productivity. In particular, digital technology is an essential instrument for advancing the modernization of CDM (Cresswell et al., 2021; Sheikh et al., 2021). Currently, digital technology has many advantages in the health field, including strengthening the medical system, improving the quality of medical services and management, assisting doctors in clinical decision-making, monitoring patients' health signs, supporting patient self-management, promoting data sharing between medical institutions and meeting the individual needs of patients (Varsi et al., 2019; Gustafson et al., 2021; Samal et al., 2021; He et al., 2022; Segal et al., 2022).

The World Health Organization and Chinese Government launched an eHealth and Internet+ Medical Health strategy in order to address the pressures of change and chronic diseases and to shape a people-centered, integrated, and digitally empowered health system (World Health Organization, 2015; Kadakia et al., 2021; WHO, 2021; Schulte and Bohnet-Joschko, 2022). Therefore, policymakers must plan for support systems, incentives, and standard setting for CDM.

### Prevalence of chronic disease

The prevalence of chronic diseases and the presence of two or more chronic conditions in an individual are on the rise (Boyd and Fortin, 2010; Ayanlade et al., 2019). Prevalence rates suggest that, globally, one in three people live with multimorbidity, with rates of 65% in people aged >65 years and 85% in people aged >85 years and rising (Doyle et al., 2021). The number of hypertensive patients worldwide rose from 594 million in 1975 to 1.28 billion in 2019 (Zhou et al., 2021). According to the Global Diabetes Atlas, there will be 536 million diabetic patients worldwide by 2021 and 783 million by 2045; there are currently 140 million diabetic patients in China, and that number will surpass 174 million by 2045 (Sun et al., 2022). The proportion of disease burden attributable to noncommunicable diseases and injuries increased from 21% of the total burden in 1990 to 34% in 2019. From 1990 to 2019, the five major diseases that led to an increased global disease burden include ischemic heart disease, diabetes, stroke, chronic kidney disease, and lung cancer (Murray et al., 2020; Roth et al., 2020; Vos et al., 2020). Chronic diseases are characterized by their long duration, high prevalence, need for closed-loop management, strong compliance, multi-disease co-occurrence, and high convenience requirements. Theoretically, the digital and integrated health management model conforms to the characteristics of chronic diseases, can alter the fragmentation of CDM, realize closed-loop management of the entire process, and facilitate effective communication and information sharing.

### Major problems in current chronic disease management

The barriers of CDM are as follows: first, there is a lack of awareness regarding the use of information technology and big data for CDM, resulting in development bottlenecks, and “technology + mechanism” has not been used to improve the quality and efficiency of CDM. Second, informational continuity and managerial continuity are two routes to effective CDM (Lionis et al., 2019; Belizan et al., 2020). However, information is fragmented between preventive and medical services, and primary care clinics and secondary and tertiary hospitals lack integration. Digital tools are not yet fully utilized to create an efficient closed-loop for CDM (Lugo-Palacios et al., 2019). Third, although the World Health Organization has proposed a person-centered framework for integrated health services, CDM is still focused on individual diseases, and communities find it difficult to transition to “person-centered” health management. Fourth, most blood pressure, blood sugar, blood lipids, and other indicators are measured using traditional methods that do not employ digital technology to achieve accurate detection and are not standardized. In addition, test results are not saved, integrated, or shared promptly, making it impossible to track information and effectively manage patients. The fifth barrier is the low rates of treatment and awareness among patients with chronic diseases. Based on a study involving 1.7 million Chinese adults, hypertension awareness and control rates were 36 and 6%, respectively. Meanwhile, according to a national study involving 1.4 million Chinese participants, 44% of those with diabetes in China were aware of their diagnosis, and only 39% were receiving treatment (Yuanfeng et al., 2021).

### The purpose of the study

Previous studies have reported the health management model, including Community health management models in the United States (Chronic Care Model, CCM) framework, Finland (Vartiainen, 2018), the United Kingdom, Germany, and Japan. The models described earlier clarify the management targets, subjects, processes, specific practices, and safeguards, but lack digitally driven CDM and do not maximize the benefits of digital technology (Zens et al., 2020). Digital technology is a key enabler for integrated models of community-based primary health care, but little is known about how existing technologies can be utilized to support the delivery of integrated care. In conclusion, as far as we know, research on the precise and digitized community health management model is scant.

This study has only considered one objective: to build Integrated Community Multimorbidity Care Model (ICMCM) based on digital technology. Our objective is to serve as a model for other regions by transitioning from traditional crude community-based CDM to refined management, from qualitative management to quantitative management, and from administrative directives to data standard-oriented management via a digitally driven system.

## Materials and methods

This study synthesizes the views of experts, service providers and service demanders to establish ICMCM, with data as its core and digital technology as its implementation pathway (Steele Gray et al., 2018). The model was built to cover experts in a number of fields, with the following structure: administrative officials from the Shanghai Municipal Health Commission and district-level health commissions; public health experts from the Shanghai Municipal Center for Disease Control and Prevention (CDC) and district-level CDCs in Shanghai; big data information companies; clinical experts from hospital; general practitioners and nurses from community health service centers; and metrologists from the Shanghai Measurement Association, all of whom have extensive experience in the field of chronic diseases. The demanders originated from community health service centers. Data collection methods are derived from big data platforms in Shanghai, including General Practitioner Contract Platform, Integrated Community Multimorbidity Management System and Municipal and District-Level Health Information Comprehensive Platform. The approach to data integration is through big data association rules that integrate data into a unified data platform through unique identifiers.

We set out the essential elements and components of the ICMCM. Based on the model, we established a data collection, integration, management, and visualization system for chronic diseases. We describe the primary modules and functions of the ICMCM, the operational processes, data integration, and initial implementation results. Moreover, we describe in detail the role of digital technology in the practice and organizational environment of ICMCM (Qaffas et al., 2021). We utilized digital, integrated, and precise technologies to manage chronic disease patients in the community. The ICMCM was piloted and promoted in Shanghai's community health service centers.

### Ethics approval

No human or animal information and data were used in this study and therefore no ethical approval was required.

## Results

### General framework of ICMCM

The connotation, basic condition, methods and tools, innovation points, management core, service providers, target group, service contents, implementation pathway and display format of the ICMCM are as follows (Table 1). First of all, we use digital devices to capture patient information; Secondly, the ICMCM is driven by cloud computing, big data, the internet of things, mobile internet, and artificial intelligence, integrating online/offline and in-hospital/out-of-hospital medical resources with digital technology to form integration of online/offline and in-hospital/out-of-hospital medical resources. Thirdly, we established a standard service and management procedure using digital technology and chronic disease service specifications. Based on the characteristics of chronic diseases, a standardized core index system is established and dynamically displayed using visualization software, enabling real time observation of the patient's health status. The primary characteristics of the new model are disease-centered to people-centered, from single fragmented services to integrated services, from extensive management to refined management, and from data information fragmentation to data interconnection.

**Table 1:** Content of ICMCM.

**Indicators**	**Contents**
Model connotation	Realizing people-centered multimorbidity prevention and management
Basic condition	Big data
Methods and tools	Digital technology
Innovation points	A mechanism for innovation for digital technology plus chronic disease services
Display format	Visualization software
Pathway	For better implementation ICMCM, chronic disease management center was established in the community health service center. The chronic disease management center is an independent area within a community health service center, and it has a standardized decor, logo, computer, and precision measuring equipment. Realized environment standardization, hardware standardization, software standardization, process standardization and information standardization
Management core	Digitization, integration, and precision
Service providers	The GP team in the community health service center, consisting of GP, physician assistants, nursing staff, and volunteers
Service target group	Chronic disease patients, covering hypertension, diabetes, stroke, chronic obstructive pulmonary disease, and cancer patients
Management purposes	Realize chronic disease management from macro to concrete, from qualitative to quantitative; enhancing the quality of chronic disease management for better health
Service contents	disease-related risk factor information registration; provide standardized height and weight, waist and hip circumference, accurate blood pressure, blood glucose measurement, colorectal cancer detection, COPD service, etc. regular screening for high-risk groups; artificial intelligent voice follow-up; treatment of chronic diseases; two-way referral; guidance for residents to use tools such as APPs to carry out independent management; targeted and universal health education

The main providers of CDM services are general practitioner (GP) teams in community health centers; patients with hypertension, diabetes, stroke, chronic obstructive pulmonary disease, and cancer are the primary populations served. The service's primary components include chronic disease risk factor registration, early warning monitoring, comprehensive assessment, health education, follow-up visits, accurate blood pressure, accurate blood sugar, height and weight, and waist and hip circumference measurements.

Implementing data, service, and technology integration using digital technology is central to ICMCM. Consequently, we implemented several interventions. Data integration refers to the integration of fragmented electronic health records, electronic medical records, and public health service databases, as well as the creation of a two-tiered urban health information platform. Horizontal integration of services refers to the integration of disease prevention, clinical treatment, risk assessment, and follow-up management; vertical integration connects the services of community health service centers, general medical institutions, specialty hospitals, and rehabilitation hospitals, and clarifies the functional positioning of different institutions in ICMCM. Technology integration refers to the implementation of technical specifications and standards for integrated health management, such as comprehensive chronic disease risk assessment, joint screening, co-morbidity follow-up, and comprehensive assessment for patients with multiple chronic diseases, as shown in Supplementary Figure S1.

### Digital technology drives data integration

We must first understand which health information platforms and data types currently exist before we can integrate information systems. In order to avoid data fragmentation and promote the implementation of the ICMCM, Shanghai has established an integrated chronic disease management information system based on electronic health records, as shown in Table 2 and Supplementary Figure S2. We integrate the database of communities, hospitals, CDCs, and wearable devices for residents using digital technology and unique identifiers. The data of chronic disease patients are stored in the integrated chronic disease management information system, which ensures that preventive care and consultation records of chronic disease patients are seamlessly accessible at different levels of competence, providing family physicians with information on lifestyle management, referral, and health care services as well as a reference basis for clinicians to develop medication and additional treatments. The modules and functions of integrated chronic disease management information system are following.

**Table 2:** The modules and functions of integrated chronic disease management information system.

**Modules**	**Function**
The functions in community health center	•First, the community health center medical staff screened patients using techniques for accurate prediction of hypertension and diabetes. Covering the entire management process of screening, intervention, management, and treatment •Secondly, Patient demographic information, medical history, screening results, and physical examination are registered and updated through integrated chronic disease management information system, then comprehensive health interventions are given based on the screening results. Allows clinicians to track patient health information over time across visits and when patients seek care at different facilities to reduce unnecessary checks and data entry
The functions in hospitals	Clinicians provide further diagnosis and treatment for patients with chronic diseases based on their screening results and provide patients with evidence-based treatment recommendations for medications, care, and lifestyle. Clinician's diagnosis, test and examination, medication and other information are synchronized and uploaded to the cloud, and family doctors can also view the relevant records
The functions in CDC	The municipal chronic disease service has access to all information of the patient, and the chronic disease information is linked to the electronic health record, enabling the integration of information. CDC provides authenticity, uniqueness control, and data analysis and statistics functions, and realizes quality management of service and data flow. Based on the results of the data review develop policy recommendations for prevention and control
Resident health management	Provide personalized health management programs to support residents' self-management by combining their overall health status. Based on individual chronic disease health management profiles, integrated chronic disease management information registration and integrated risk assessment functions to alert residents of their health status. Residents can register, follow-up and give feedback on the quality of services online
Comprehensive display	A comprehensive display of the current status and effectiveness of chronic disease work, as well as the incidence, trends, and management of chronic diseases

Shanghai has established the General Practitioner Contract Platform, Integrated Community Multimorbidity Management System and Municipal and District-Level Health Information Comprehensive Platform, enabling the interconnection and sharing of data between the three platforms. This study combines data from community health service centers with diagnosis and treatment data from secondary and tertiary hospitals to achieve precise targeting and closed-loop management of chronic disease services in the community. After data integration, we can observe the following primary data: Shanghai GP contract data sharing, cross-regional and cross-organization clinical data automatic access, suspected disease population alert, chronic disease comprehensive risk assessment, chronic disease co-morbidity management, etc.

Alternatively, using big data capture and matching, identify residents at high-risk of chronic diseases, remind doctors to provide screening services in the clinic or at an elective date, and automatically track clinical diagnosis information, prompting doctors to include it in patient management, thereby forming closed-loop management of chronic disease screening services. For CDM objects, the system automatically tracks the diagnosis and detection information of a variety of chronic diseases and complications, prompts physicians to adjust the management requirements of comorbidities based on abnormal indicators and emerging diseases, and integrates clinical and preventive services in between consultations. Based on vertical integration, provide continuity of care for patients with chronic diseases and improve the integration and sharing of medical and preventive information, as shown in Supplementary Figure S3.

### Digital technology drives accurate measurement

First, the digitization of infrastructure and equipment. The chronic disease management centers were established within the community health service center to better implement the ICMCM and exclude environmental interference during the examination of patients with chronic diseases. The chronic disease management center is an independent area within a community health service center, and it has a standardized decor, logo, computer, and accurate measurement equipment. For the management of chronic diseases, we use precise and digitally-advanced testing equipment. The chronic disease management center is outfitted with accurate blood pressure measurement equipment, accurate blood glucose measurement equipment, a portable pulmonary function meter, an accurate height, and weight measuring instrument, an accurate waist and hip circumference detector, intelligent voice follow-up information collection equipment, and colorectal cancer screening facilities, chronic obstructive pulmonary disease detection; the intelligent follow-up booth supports intelligent human-machine voice interaction. The chronic disease management center installed triage systems, evaluation and display large screens, computers, identification, and additional information equipment.

Second, consistent and precise services. Community Health Management Work Practices—Integrated Prevention and Treatment of Chronic Diseases Community health care workers take precise measurements following service requirements. Provide accurate blood pressure measurement services for hypertensive patients and accurate glucose measurement services for diabetic patients to eliminate measurement errors and increase the convenience and accessibility of these services for residents.

Thirdly, digitalization technologies. A comprehensive risk assessment is performed using digital technology that captures the patient's basic personal information, family history, disease history, medication history, lifestyle, and major test results. Utilization of appropriate technologies for integrated disease prevention and control to screen chronic disease patients. GP and intelligent voice follow-up device based on monitoring of key symptom indicators, complication screening, and co-morbidity follow-up, as shown in Supplementary Figure S4.

### Digitally driven management

For the visualization of ICMCM, we have developed a visualization display platform that shows the status of quantity, quality and effectiveness of ICMCM. Visualization technology can create a digital re-enactment of the scene so that people can more easily understand the issue. Currently, a new index system of ICMCM has been established. It consists of seven modules, 42 sub-modules, and 121 specific indicators of health registration, chronic disease monitoring, early detection, and ICMCM effect, quality control, and standardized service level, all of which are included in the Integrated Chronic Disease Management Information System Platform. Moreover, we develop a systematic understanding of ICMCM through the cross-combination and evaluation of ICMCM indicators to provide a foundation for enhancing the comprehensive ICMCM and evaluation mechanism and enhancing its efficacy. The visualization of ICMCM is as shown in Supplementary Figures S5, S6.

## Discussion

Based on information technology, we developed ICMCM and provided services and management for patients with chronic diseases according to ICMCM. The ICMCM was utilized to create health records and conduct comprehensive risk assessments for 1,975,100 residents, provide health education and screening for 1,492,900 high-risk subjects, and manage 1,816,500 patients. More than 6.5 million times of pertinent diagnosis and treatment information of secondary and tertiary medical institutions for patients with chronic diseases have been pushed, creating a closed-loop of health management services. Establish three community support centers for chronic disease health management, provide standardized health management services to 260,000 residents, and offer online app inquiry services to 400,000 people. Eighty-nine communities can apply standard measurement technology for health management, achieving “people-centered” integrated and accurate management of a variety of chronic diseases.

There are a variety of approaches to CDM at present, and we summarize the characteristics of this study and other approaches to CDM. In Finland, the North Karelia Project was initiated in 1972 as a population-based intervention focusing on the reduction of public health risk factors and changes in lifestyle. Some examples include lowering cholesterol with a low-fat diet, controlling blood pressure with a low-salt diet, and quitting smoking. The project disseminated health education materials to the populace through the media to bolster grassroots efforts (Jousilahti et al., 2016; Puska and Jaini, 2020). To prevent and control chronic diseases, Japan adopted a model in 2002 that combines specific health screening and specific health guidance. All insured individuals between the ages of 40 and 74 receive specific health examinations, are classified into low-risk and high-risk groups based on the results of the examinations and their lifestyle habits, and are provided with specific health advice (Xiaoli, 2010). The National Health Service policies guarantee CDM in the UK, and community chronic disease prevention and treatment is primarily carried out by GPs, community nurses, and social workers, with strict implementation of community GP consultations, appointments, and two-way referral mechanisms between major hospitals, and a comprehensive health information system; patients are assisted by health care professionals in carrying out some preventive or curative health care activities (Shaw et al., 2015).

The CCM is a model of care that designs the essential elements of chronic disease care, such as a health system or organization, clinical information systems, decision support, delivery system design, self-management support, community organizations, and patient resources. World Health Organization proposed innovative care for chronic conditions, which includes supporting a paradigm shift, managing the political environment, building integrated health care, aligning sectoral policies for health, using healthcare personnel more effectively, center care on the patient and family, supporting patients in their communities, and emphasize prevention. The model acknowledges the broader policy environment that includes patients, their families, healthcare organizations, and communities (Grover and Joshi, 2014).

Compared to the aforementioned models of health management model, we have the following advantages. First, we attempt to develop ICMCM that corresponds to the characteristics of the digital era, is compatible with the long-term, multi-disease, complex, lifelong medication and other characteristics of chronic diseases, and is compatible with the medical and health system mechanism. Now that the digital age has arrived, CDM models must also adapt to the growth of productivity. Second, we have made full use of big data technology integration to achieve interconnection and sharing of databases for ICMCM and clinical diagnosis and treatment; provide accurate and standardized services for patients to ensure homogeneity of services and reliability of data; use artificial voice intelligence technology to follow-up on patients; use advanced technology to provide robust support for ICMCM. Third, one of the highlights of this model is the perfect combination of “technology + system” which converts complex data into cognition and visualization, provides a comprehensive evaluation of ICMCM via the cross-combination of multidimensional indicators, and directs the rational allocation of resources. Finally, we transform passive management into active management, enhance management efficiency, and realize the closed-loop management of chronic diseases.

Indeed, our research is not without flaws. For example, the ICMCM is being piloted and rolled out gradually in Shanghai. The digitally driven ICMCM has high requirements for information foundation and precise equipment, and economically disadvantaged regions may need to invest more resources.

We hope that the ICMCM will provide lessons for CDM elsewhere. We will evaluate the implementation effect of the model using the theoretical knowledge of implementation science. Moreover, we will continue to explore the causal mechanisms underlying the health effects of ICMCM.

## Data availability statement

The original contributions presented in the study are included in the article/Supplementary material, further inquiries can be directed to the corresponding authors.

## Author contributions

MC and MS contributed to the conceptualization and study design. SZ, YW, and QYan performed the literature review. QYang, FW, and LX analyzed the data and wrote the draft. YS and CF contributed to review. All authors contributed to the article and approved the submitted version.
