# Supplementary material for: The digitized chronic disease management model: scalable strategies for implementing standardized healthcare and big data analytics in Shanghai
Source: Front Big Data. 2023 Aug 24;6:1241296. doi: 10.3389/fdata.2023.1241296 (PMC10483282; doi:10.3389/fdata.2023.1241296)
Supplement: Supplementary file 1 [file Data_Sheet_1.docx]

**Supplement Material**


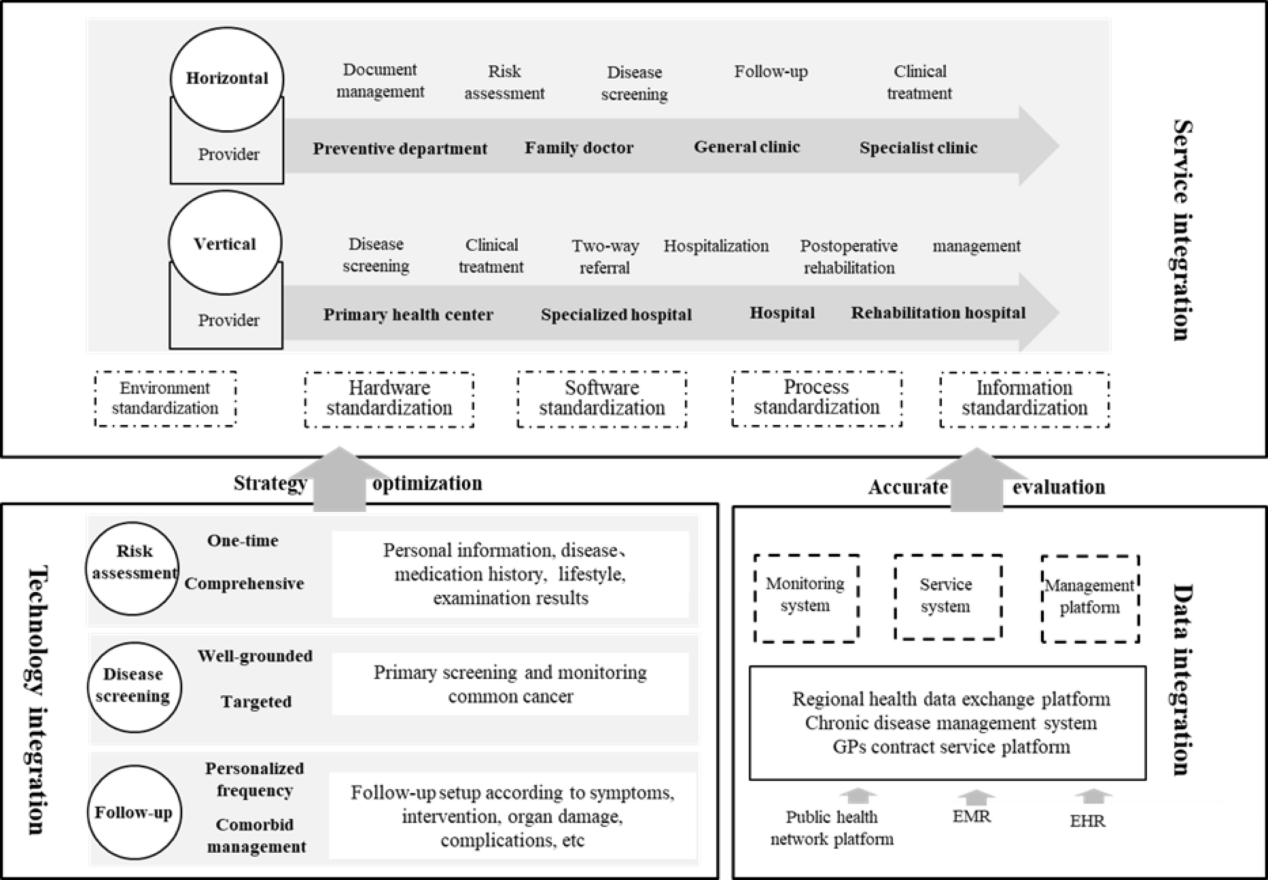


**Fig S1. Integrated Community Multimorbidity Care Model**


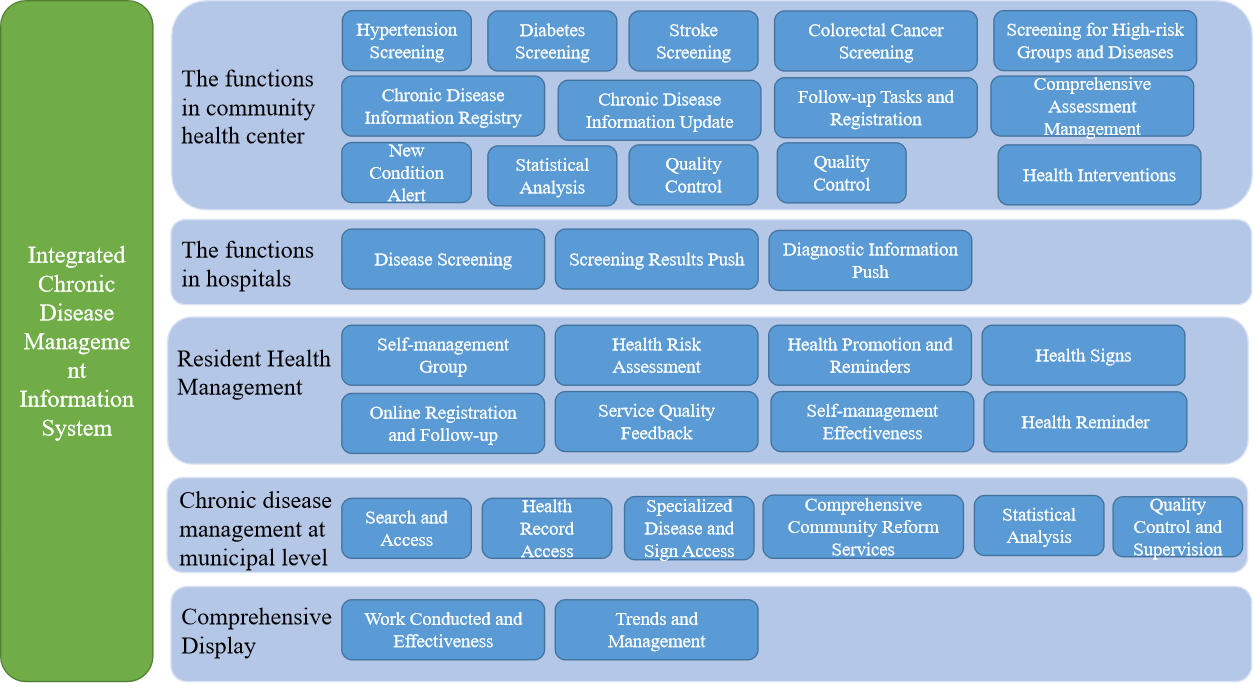


**Fig S2. The Modules of Integrated Chronic Disease Management Information System**


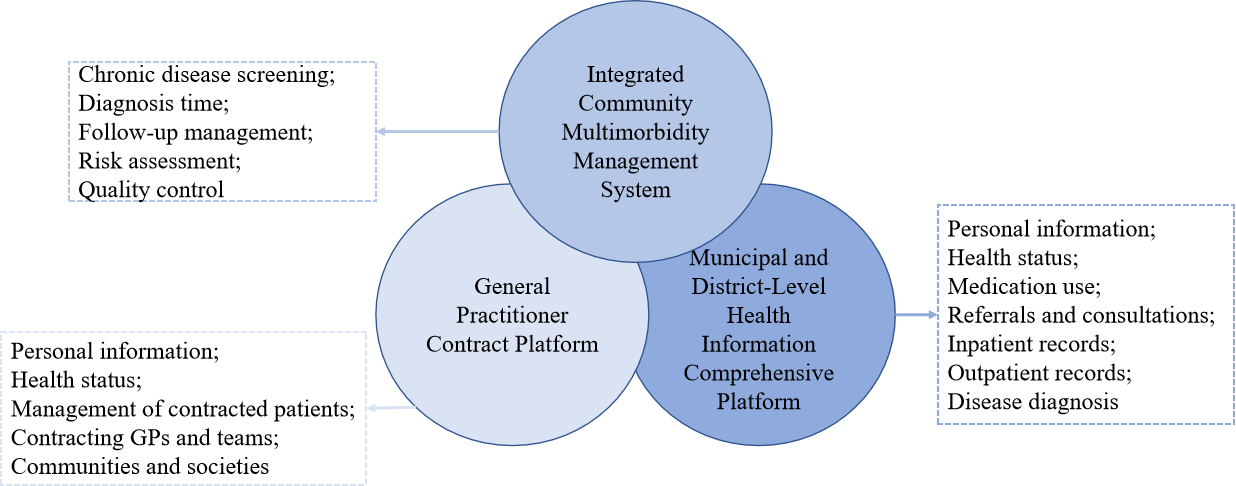


**Fig S3. Integration of data from different information platforms**


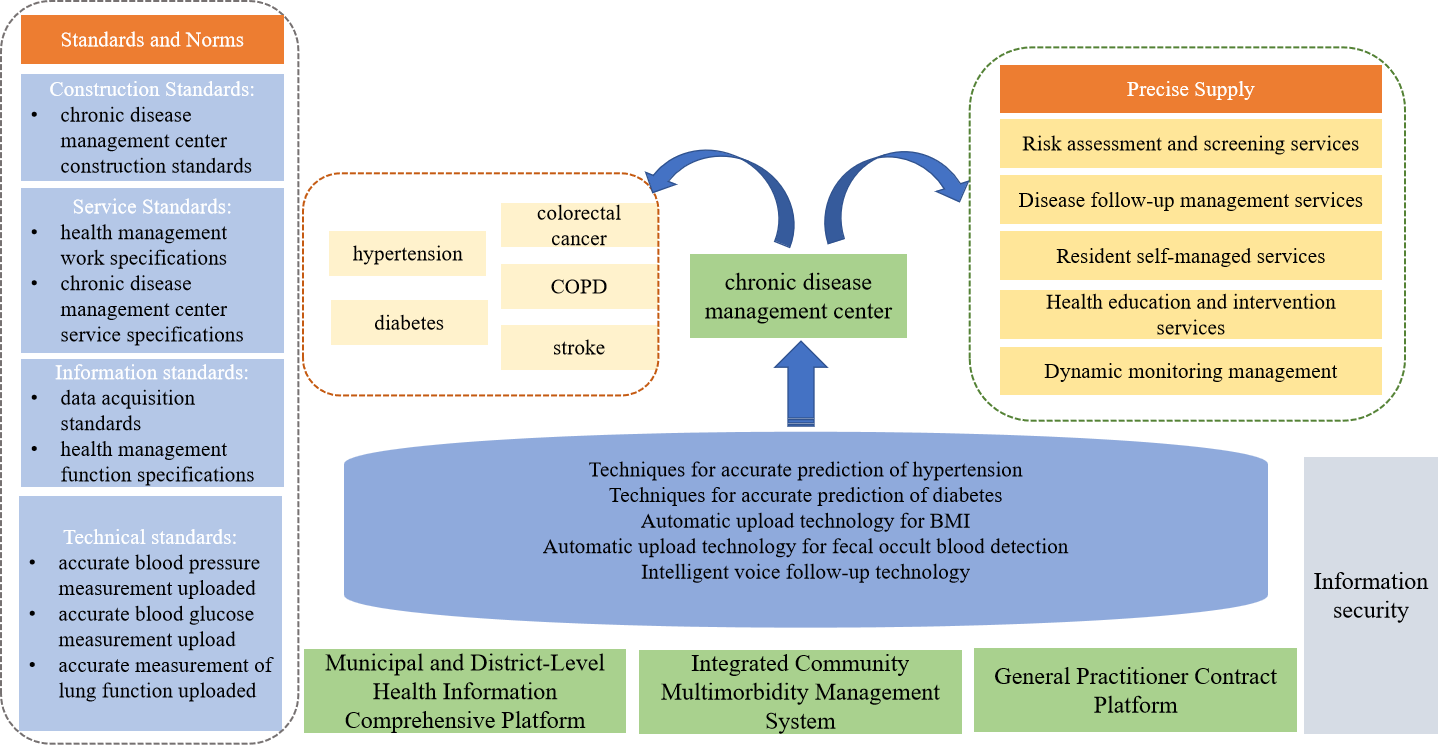


**Fig S4. Accurate measurement driven by digital technology**


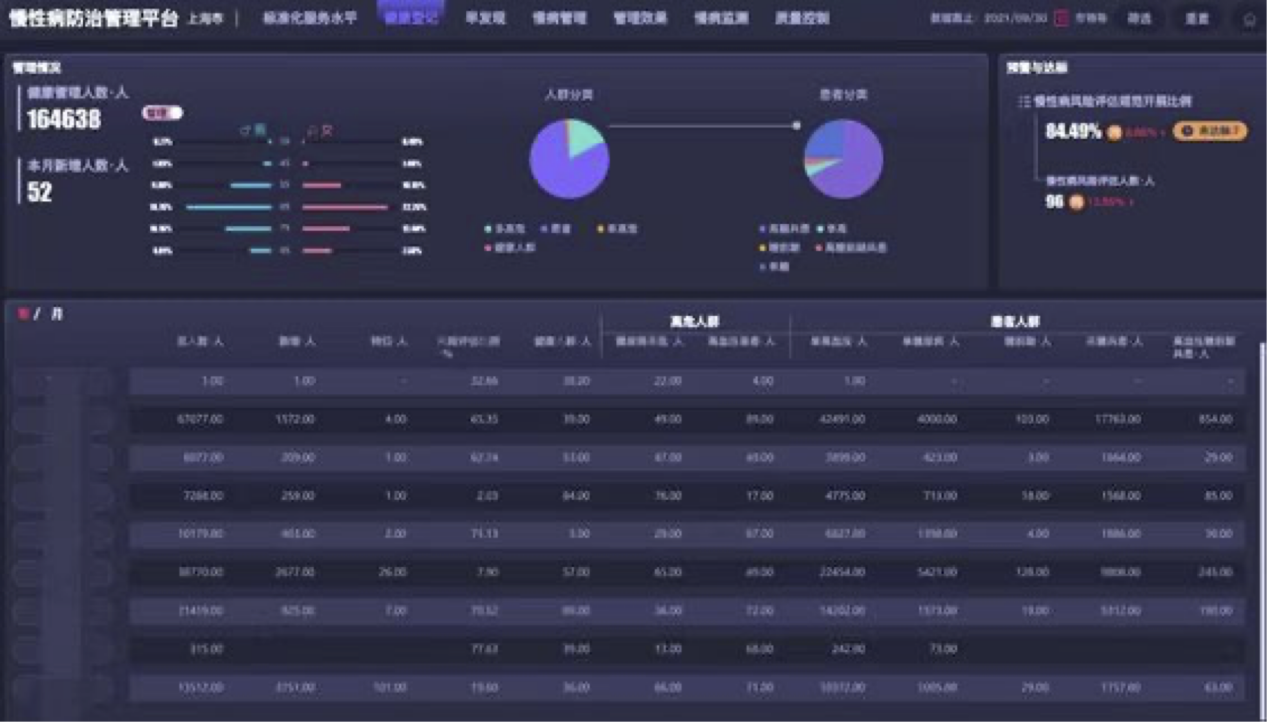
 **Fig S5. Visual display of ICMCM (1)**


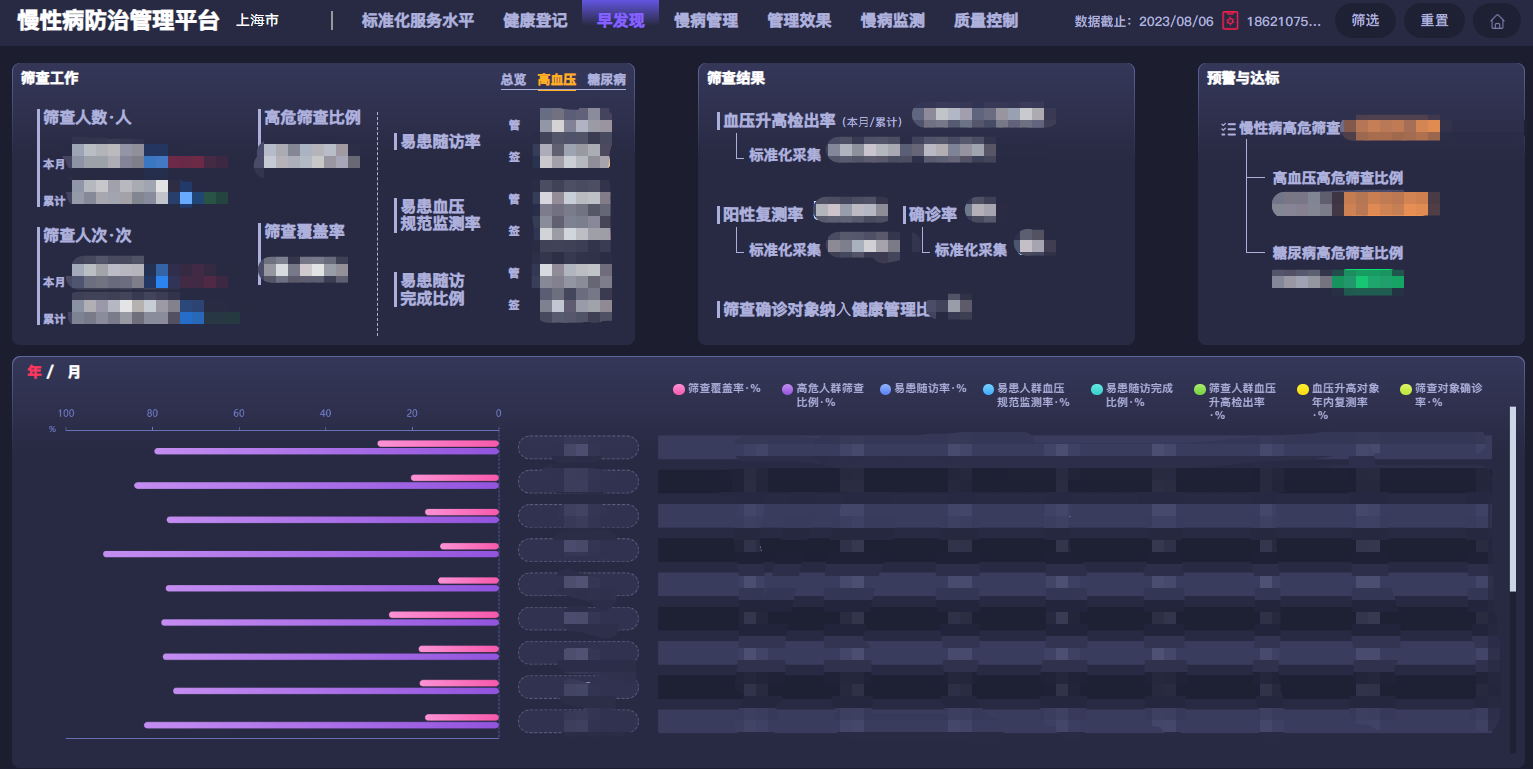


**Fig S6. Visual display of ICMCM (2)**
